# Cortical and subcortical anatomy of chronic spatial neglect following vascular damage

**DOI:** 10.1186/1744-9081-4-43

**Published:** 2008-09-22

**Authors:** Laetitia Golay, Armin Schnider, Radek Ptak

**Affiliations:** 1 Division of Neurorehabilitation, Geneva University Hospitals and University of Geneva, 1211 Geneva 14, Switzerland; 2 Fondation Plein Soleil, 1010 Lausanne, Switzerland

## Abstract

**Background:**

The role of the inferior parietal lobule (IPL) and superior temporal gyrus (STG) or subcortical pathways as possible anatomical correlates of spatial neglect is currently intensely discussed. Some of the conflicting results might have arisen because patients were examined in the acute stage of disease.

**Methods:**

We examined the anatomical basis of spatial neglect in a sample of patients examined in the post-acute stage following right-hemispheric vascular brain damage. Lesions of 28 patients with chronic spatial neglect were contrasted to lesions of 22 control patients without neglect using lesion subtraction techniques and voxel-wise comparisons.

**Results:**

The comparisons identified the temporo-parietal junction (TPJ) with underlying white matter, the supramarginal gyrus, the posterior STG, and the insula as brain regions damaged significantly more often in neglect compared to non-neglect patients. In a subgroup of neglect patients showing particularly large cancellation bias together with small errors on line bisection damage was prevalent deep in the frontal lobe while damage of patients with the reverse pattern was located in the white matter of the TPJ.

**Conclusion:**

Considering our results and the findings of previous studies, spatial neglect appears to be associated with a network of regions involving the TPJ, inferior IPL, posterior STG, the insular cortex, and posterior-frontal projections. Frontal structures or projections may be of particular relevance for spatial exploration, while the IPL may be important for object-based attention as required for line bisection.

## Background

Damage to the right cerebral hemisphere may lead to a severe impairment in spatial orienting towards the left side of space: spatial neglect [1,2]. The exact locus of damage leading to this striking and debilitating disorder is currently intensely discussed [3-6]. Early investigations, based on visual inspection of computerized tomography (CT) scans, suggested that the inferior parietal lobule (IPL) and the temporo-parietal junction (TPJ) are the brain regions most often damaged in patients with spatial neglect [7,8]. However, these studies may be criticized on the ground of their inclusion criteria – in particular, neglect may have been confounded with visual field loss – as well as the absence of a comparison between patients with and patients without neglect. Nevertheless, many authors affirmed that among the cortical structures important for spatial orienting, the IPL plays a crucial role [2,9-11]. It was therefore surprising that a recent study comparing 25 neglect to 25 control patients, reported that the region best predicting the occurrence of spatial neglect was the superior temporal gyrus [STG, [12]]. This study used a new lesion subtraction technique, which attempts to identify the brain regions most strongly associated with spatial neglect by subtracting the overlaid lesions of non-neglect patients from the lesions of neglect patients [13]. However, the role of the STG for spatial awareness was called into question by several authors, who objected that the authors had excluded patients with visual field loss, which might have biased their analysis in favour of more anterior damage [5,14]. In order to examine the anatomy of neglect in a prospective study, Mort et al. [14] performed high-resolution MRI on 14 neglect patients with vascular damage in the territory of the middle cerebral artery. In significant contrast, these authors found that the region predicting best the occurrence of neglect was the angular gyrus. However, Karnath et al. [15] confirmed their initial results in a second study on a very large sample (n = 78 neglect patients, n = 62 controls), which did not exclude neglect patients with visual field loss.

Several factors other than differences in sample size and statistical power could explain these conflicting results. One important difference was the time of examination respective to lesion onset. Whereas in the study of Mort et al [14], patients were evaluated 63 days post-stroke, Karnath et al. [15] examined neglect patients very early (median: 8 days) following their brain injury. However, as most of the patients exhibiting severe spatial neglect in the first week post-stroke recover within the following three months [16,17], evaluating the anatomy of spatial neglect in acute patients may lead to underestimation of brain regions involved in long-lasting impairments [18]. Also, different criteria for patient selection might have influenced the anatomical findings. Thus, some patients in the study of Mort et al. [14] manifested neglect only in line bisection, but showed normal spatial exploration. Spatial neglect is not a unitary syndrome, and some behavioural manifestations of neglect might be associated with different brain damage than other symptoms [19].

The present study evaluated the frequency of damage of the IPL and STG in 50 patients with right-hemispheric vascular damage, 28 presenting spatial neglect two months post-injury. In order to avoid a selection bias in our sample, we did not apply any *a priori *anatomical or etiological exclusion criteria. In addition, we evaluated brain lesions of a subgroup of neglect patients particularly impaired in line bisection with patients particularly impaired in spatial exploration and found evidence for an anatomical dissociation between these two groups of neglect patients.

## Methods

### Participants

50 patients with recent vascular damage to the right cerebral hemisphere admitted to our rehabilitation service were studied. All patients gave written consent according to the Declaration of Helsinki, and the study was approved by the Ethical Committee of the University Hospital Geneva. We included patients with a recent unilateral brain injury, the only exclusion criteria being the presence of previous brain damage or absence of a visible brain lesion on MRI or CT. The presence of motor and somatosensory impairment was assessed using standardized neurological examination. Visual fields were examined using standard confrontation testing. Patients were attributed to the neglect group if they a) showed at least some signs of contralesional unawareness in everyday actions (e.g. ipsilesional head and gaze deviation, difficulty with dressing or grooming due to personal neglect, unawareness of objects or people placed contralesionally etc.) and b) had scores indicative of spatial neglect in at least two of three formal neglect tests. The three tests used to assess neglect were the following:

#### "Bells test" [20]

participants were required to cancel out small bells dispersed among other symbols printed on a horizontally presented sheet of A4 paper. Five or more (of 15) contralesional omissions were considered as a sign of spatial neglect [20].

#### Line bisection [21]

patients marked the middle of 18 lines ranging from 12–20 cm aligned horizontally on an A4 sheet of paper. The deviation from true midline was calculated in percent of the line length. A mean ipsilesional bias of more than 10% differentiated the patients' score significantly (significance level: α < .05) from the mean of neurologically healthy participants [21], and was therefore considered indicative of spatial neglect.

#### Drawing [22]

patients were asked to copy simple forms (star, clock, flower). Their drawings were rated with respect to the presence of contralesional omissions, with 0 indicating absence of elements on the left, 1 lack of precision or distortion of the left side, and 2 a complete drawing. Scores below 2 were considered consistent with spatial neglect.

Using these criteria, 28 patients were attributed to the neglect group and 22 patients to the control group. The clinical characteristics of these two patient groups are shown in Table 1. Patients were tested at a post-acute stage of disease and scans were made on the average more than one month after onset. The two groups did not differ with respect to age [t(48) = .79, n.s.], time since injury [t(48) = 1.01], time of scan [t(48) = .11], or frequency of limb paresis [Fishers exact test, n.s.]. In contrast, there was a significant difference between groups in all three neglect measures: cancellation [t(48) = 11.03, p < .0001], line bisection [t(48) = 4.78, p < .0001], and drawing [Wilcoxon test, p < .0001]. The groups also differed with respect to the frequency of visual field defects: in the neglect group, 4 patients had hemianopia and one patient left inferior quadrantanopia, while one patient of the control group had left hemianopia.

**Table 1:** Patient description

		Neglect	Control
Number		28	22
Sex	Female/male	17/11	9/13
Age	Years, mean	66.3 (39–83)	69 (47–88)
Aetiology		19 infarction	17 infarction
		9 haemorrhage	5 haemorrhage
Time lesion-exam	Days, mean	57.5 (17–195)	74.1 (15–387)
Time lesion-scan	Days, mean	46.5 (3–478)	50 (1–470)
Contralateral paresis	N (percent)	25 (89.3%)	17 (77.3%)
Lesion volume	ccm^3^	105 (9–235)	38.9 (1–135)
Visual field defect	N (percent)	5 (17.9%)	1 (4.6%)
Bells test	Left omissions	11.5 (2–15)	0.9 (0–4)
	Right omissions	3 (0–9)	0.5 (0–3)
Line bisection	% right deviation	11.9 (-1–33)	2.3 (-1–13)
Drawing neglect	Mean	0.8 (0–2)	2 (2-2)

Demographic and clinical description of the neglect and control patients (range or percentage values indicated in parentheses).

### Lesion comparisons

Lesion mappings were based on manual transposition of lesions from individual MRI-scans or CT-scans into the template brain of the Montreal Neurological Institute (MNI). Since this is a retrospective study, we used the brain scans that were available for individual participants at the time of the clinical examination. T2-weighted MRI had been performed in 52%, T1-weighted MRI in 12% and CT in 36% of patients. The clinical MRI protocol comprised a 25-slice acquisition (slice-thickness: 6 mm). In most patients (74%) brain images were acquired at least 5 days post-injury (CT-scans, mean: 32.2 days; MRI-scans: 57 days).

In order to minimize errors of transfer from the brain scan to the template image, we proceeded in the following steps [23]: i) slices were selected that provided the best match to selected slices from the template brain; ii) individual lesion boundaries and the major sulci were identified and delineated as regions of interest (ROI) directly on digitized images of the axial brain scans; iii) the lesion ROI was transferred from the individual scan to the template slice, taking into account its relation to neighbouring sulci; iv) these adapted lesion boundaries were re-drawn on the same MNI-slices using MRIcro software [13].

MRIcro was used to generate images of superposed lesions, lesion subtractions, and voxel-wise comparisons. Lesion superposition generates an image in which the number of patients sharing a lesion in an anatomical region is colour-coded, ranging from violet (damage present in only one patient) to red (damage present in all patients). Lesion subtraction is a means to measure the frequency of brain damage for specific subregions in the neglect group after subtraction of the control group. This technique provides a relative estimate of incidence of damage specific to neglect. Voxel-wise comparisons are based on a four-cell χ^2^-test comparing the frequency of involvement (damaged, undamaged) in each group (neglect, control) independently for each voxel. The position of significant differences is indicated in x-, y-, and z-coordinates of the Talairach-space [24].

## Results

**Figure 1:**
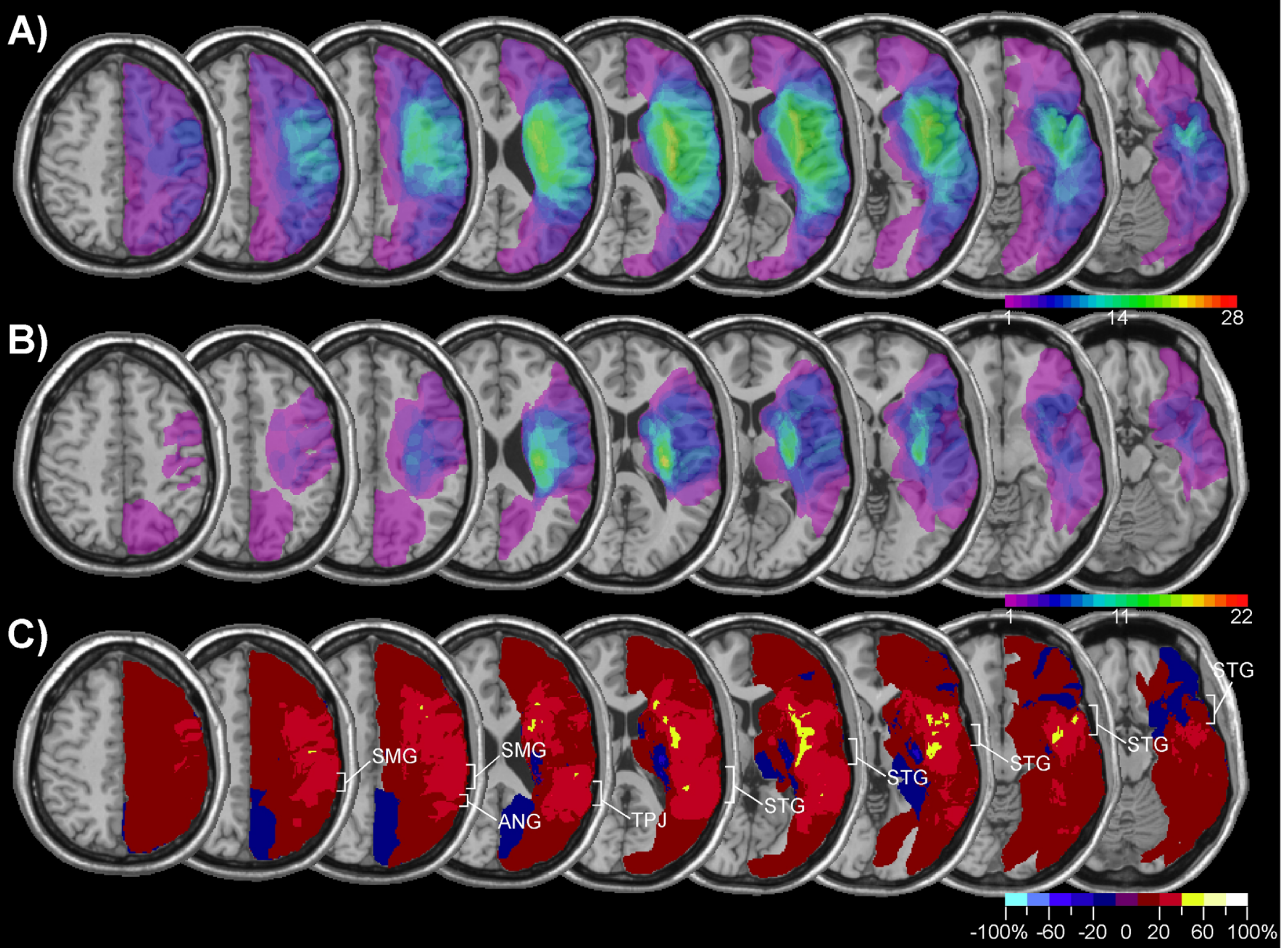
**Lesion analysis of 28 patients with spatial neglect and 22 control patients without spatial neglect**. The figure shows transverse sections of the MNI template brain with Talairach z-coordinates increasing from right (z = -16) to left (z = 48). A) Overlap map of neglect patients showing the frequency of damage for each voxel of the template brain. The colour scale indicates the increasing frequency of overlapping lesions from violet (n = 1) to red (n = 28, neglect patients; n = 22, control patients). B) Overlap map of control patients without spatial neglect. C) Subtraction plot showing colour-coded relative frequency of damage in the neglect group after subtraction of the control group. Reddish colours indicate relative prevalence of damage in the neglect group, shown in bins of 20% from dark red (1–20%) to white (80–100%). Bluish colours indicate prevalence of damage in the control group from dark blue (1–20%) to light blue (80–100%).

**Figure 2:**
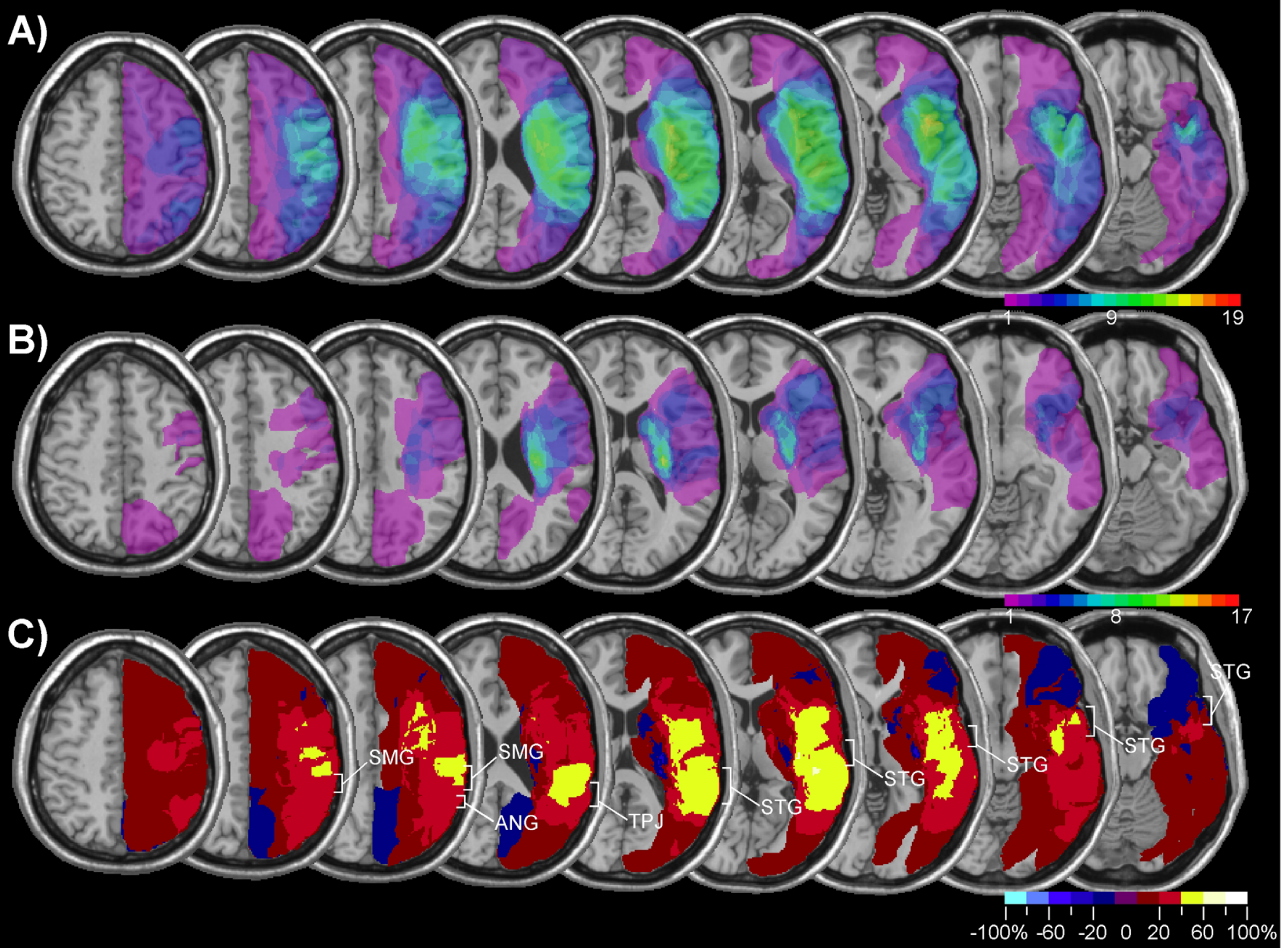
**Lesion analysis of 19 patients with spatial neglect and 17 control patients with ischemic brain damage**. Same analysis as shown in Figure 2, but confined to patients with ischemic damage. A) Overlap map of neglect patients. B) Overlap map of control patients without spatial neglect. C) Subtraction plot.

Figure 1 presents the lesion-density plots for neglect and control patients. The average lesion volume (Table 1) was about three times greater in neglect than control patients [t(48) = 3.78, p < .001]. The region that was most frequently damaged in neglect patients involved the insula and underlying white matter, the posterior temporal and inferior parietal cortex (Figure 1A). However, as can be seen in Figure 1B these regions were also frequently damaged in control patients. In order to identify regions that were more frequently damaged in neglect patients compared to the control group, we subtracted the superimposed lesions of the control group from the lesions of the neglect group (Figure 1C). This subtraction identified a region reaching from the posterior insular cortex into the white matter of the frontal lobe as predicting best the incidence of spatial neglect. However, the analysis shown in Figure 1 was not only based on patients with cerebral ischemia, but also included a significant number of patients with haemorrhagic brain damage. Though the inclusion of patients with different vascular aetiologies renders our results comparable to previous studies, the fact that cerebral haemorrhage tends to be subcortical, does not always respect vascular territories and is difficult to delineate due to oedema surrounding the lesion might have biased the lesion overlap in favour of subcortical regions. We therefore performed the same analysis, but included only patients with ischemic brain damage (Figure 2A and 2B). The subtraction map based on this comparison (Figure 2C) showed, that in comparison to patients without neglect brain damage in neglect patients was more frequent in a region surrounding the TPJ and involving the IPL, the posterior STG, and the insula (damage at least 40% more frequent in neglect compared to non-neglect patients). Note also that the region differentiating best between neglect and control patients was not confined to cerebral cortex, but reached far into the white matter beneath the TPJ and STG.

The advantage of the subtraction technique is that only regions for which frequency of involvement is different in the neglect compared to the control group are highlighted. However, the technique does not provide a statistical test of frequency of lesion involvement. In order to determine the frequency of STG and IPL damage in neglect and non-neglect patients, we determined the number of patients in each group that had damage to at least a portion of the STG or IPL. In order to demarcate the STG and IPL we used the criteria defined by Mort et al. [14]. 20 patients with spatial neglect (71.4%) and 9 control patients (40.9%) had damage to the STG while 16 neglect patients (57.1%) and only 2 control patients (9.1%) had damage to the IPL. The incidence of STG [χ^2 ^= 4.71, p < .05] and IPL damage [Fisher exact test, p < .001] was significantly higher in neglect compared to control patients. However, a limitation of this approach is that the STG has a greater anterior-posterior extension and is located more centrally within the vascular territory of the middle cerebral artery (MCA) compared to the IPL. Therefore, neglect patients may have damage to the STG simply because there is a high probability that MCA infarcts damage at least some portion of the STG. This hypothesis may be tested by computing the sensitivity (i.e. the probability that spatial neglect occurs as a consequence of damage to the region) and specificity (i.e. the probability that no neglect occurs when the region is spared) of STG and IPL damage as predictors of the occurrence of spatial neglect (Table 2). Sensitivity was slightly, but not significantly higher for the STG compared to the IPL [χ^2 ^= 1.24]. In contrast, specificity was significantly lower for the STG than the IPL [Fisher exact test, p < .02]. When patients with haemorrhages were excluded, sensitivity and specificity slightly increased (Table 2), but there was still no statistical difference between the sensitivities of STG and IPL damage to neglect while IPL damage was highly predictive of spatial neglect in patients with infarction, and more so than STG damage [Fisher exact test, p < .05].

**Table 2:** Measures of sensitivity and specificity

		STG	IPL
Infarction and haemorrhage	Sensitivity	71.4% (20/28)	57.1% (16/28)
	Specificity	59.1% (13/22)	90.9% (20/22)
			
Infarction	Sensitivity	78.9% (15/19)	68.4% (13/19)
	Specificity	64.7% (11/17)	94.1% (16/17)

Sensitivity and specificity of superior temporal gyrus (STG) and inferior parietal lobule (IPL) damage as predictor of spatial neglect.

**Figure 3:**
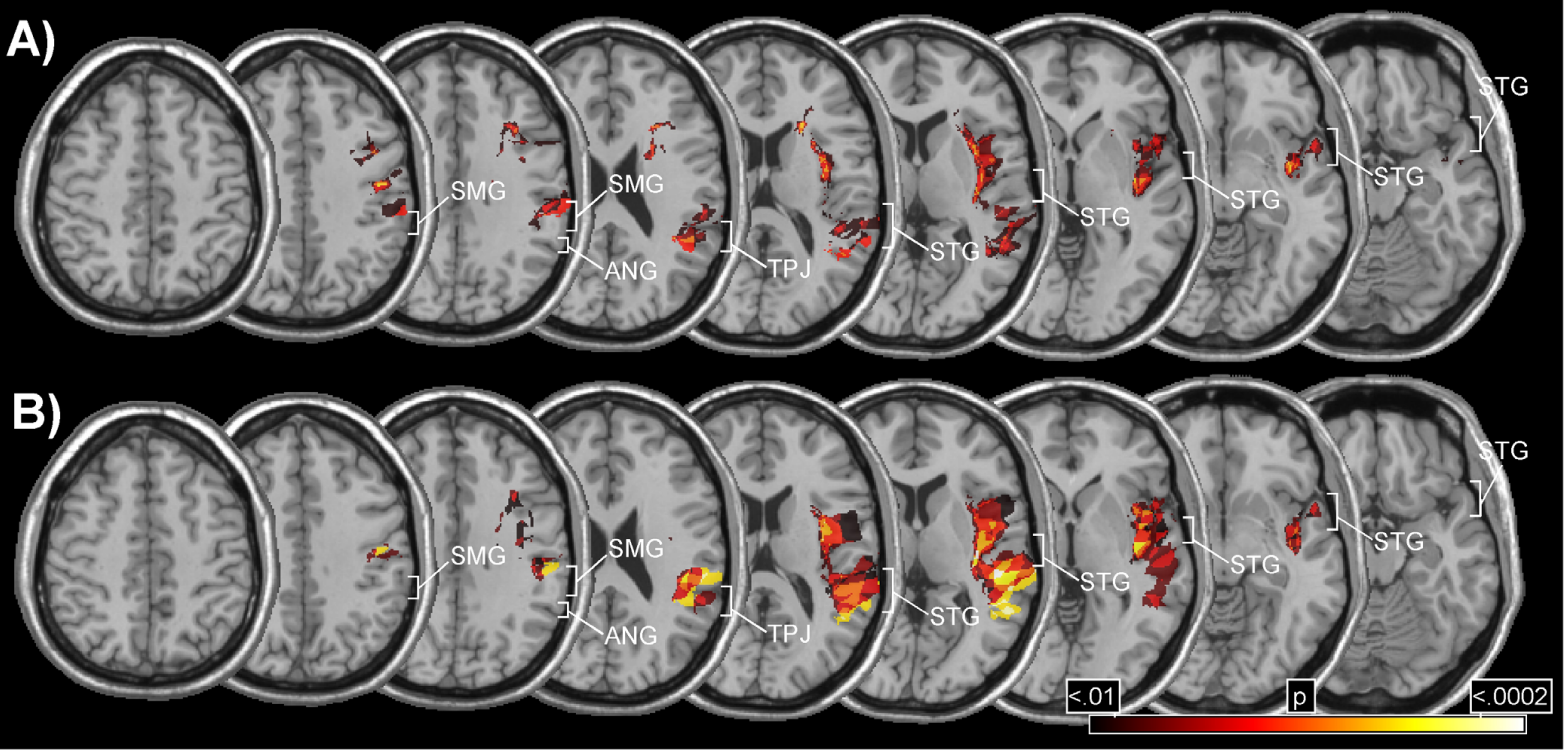
**Voxel-wise lesion comparisons**. A) Voxel-wise analysis evaluating anatomical differences between the neglect group and the control group (all patients included). B) Voxel-wise analysis including only patients with damage due to infarction. The colour codes the increasing size of the χ^2^-value from dark red (χ^2 ^= 6.63, p < .01) to white (χ^2 ^= 14.71, p < .0002).

We further evaluated the incidence of damage to specific cortical and subcortical areas with a voxel-wise analysis. In this analysis, a χ^2^-test is performed for every 'damaged' voxel testing the hypothesis whether it is involved significantly more often in the neglect group compared to the control group. In order to maximize the power of this analysis, only voxels that were damaged in at least 10 patients were examined, which in our sample resulted in 21 517 voxels tested. In view of the high number of statistical tests performed, we accepted only voxels for which the test reached a significance level of p < 0.01. This criterion was preferred to a Bonferroni-correction, which tends toe be over-conservative for very large numbers of comparisons [25]. Figure 3A displays only those voxels that differentiated with a χ^2 ^of at least 6.63 (p < .01) between neglect and control patients. All voxels shown in the figure were damaged more often in the neglect than the control group. There were essentially two clusters of voxels for which the test was statistically significant, an anterior and a posterior cluster. The anterior cluster reached from the inferior to the superior insular cortex and further into the white matter of the frontal lobe anterior to the horn of the lateral ventricle. Within this cluster a group of voxels differentiating best between both groups [χ^2 ^= 13.99, p < .0002] was situated slightly anterior to the head of the caudate nucleus (Talairach-coordinates: 21,22,14). The posterior cluster was located at the TPJ and reached into the supramarginal gyrus and the inferior postcentral gyrus. The STG was also involved, but only with its most posterior part neighbouring at the TPJ. Within the posterior cluster, a group of voxels in the white matter beneath the TPJ (Talairach-coordinates: 36,-45,24) differentiated best between neglect and control patients [χ^2 ^= 12.41, p < .0005].

A voxel-wise comparison including only patients with infarction showed a similar result (Figure 3B), the notable difference to the previous analysis being that the anterior cluster reached less far into the frontal white matter. However, note that because of the smaller sample size this analysis had less statistical power than the previous comparison.

Thus, our comparison between neglect and non-neglect patients revealed an anterior and a posterior cluster differentiating best between these two groups. Both, the IPL and the STG were damaged significantly more often in neglect than in control patients, although only the posterior part of the STG was involved.

**Figure 4:**
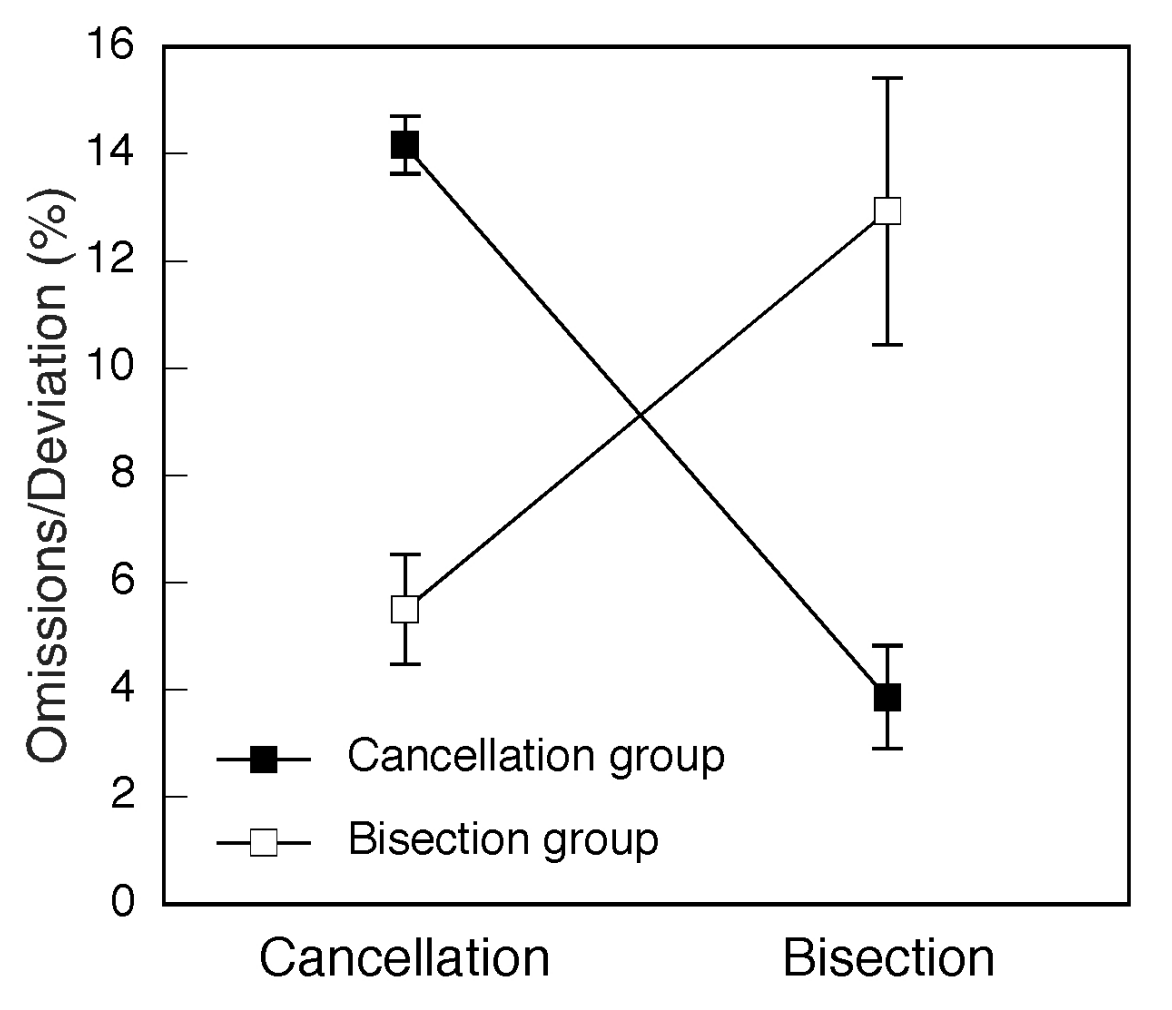
**Performance in cancellation and line bisection of subgroups of patients with neglect**. The figure shows average performance of two groups of 6 neglect patients on the cancellation (number of omissions on the left of the sheet, max. = 15) and line bisection (ipsilesional deviation of the estimated midpoint in %) tasks. Error bars show standard error of the mean.

Previous studies have suggested that patients with a strong bias on cancellation tasks may anatomically dissociate from patients with a large line bisection error. While frontal or subcortical injury has been reported in the former [19], the latter are more likely to exhibit posterior brain lesions [26,27]. These differences might explain some conflicting results regarding neglect anatomy reported in previous studies. In order to test this hypothesis, we identified in our neglect group 6 patients with a number of left omissions in the 'Bells test' that was higher than the average of the whole neglect group, but who showed bias on line bisection that was smaller than the average (group 'cancellation'). We compared these patients to 6 patients whose number of cancellation omissions was below average, but who had large biases on line bisection (group 'bisection'). The mean ipsilesional bisection bias and the number of left omissions in the cancellation test of these two groups are shown in Figure 4. The 'cancellation' group made significantly more left omissions in the 'Bells test' [t(10) = 7.88, p < .0001], whereas the 'bisection' group had a significantly larger ipsilesional bias on line bisection [t(10) = 3.38, p < .01].

**Figure 5:**
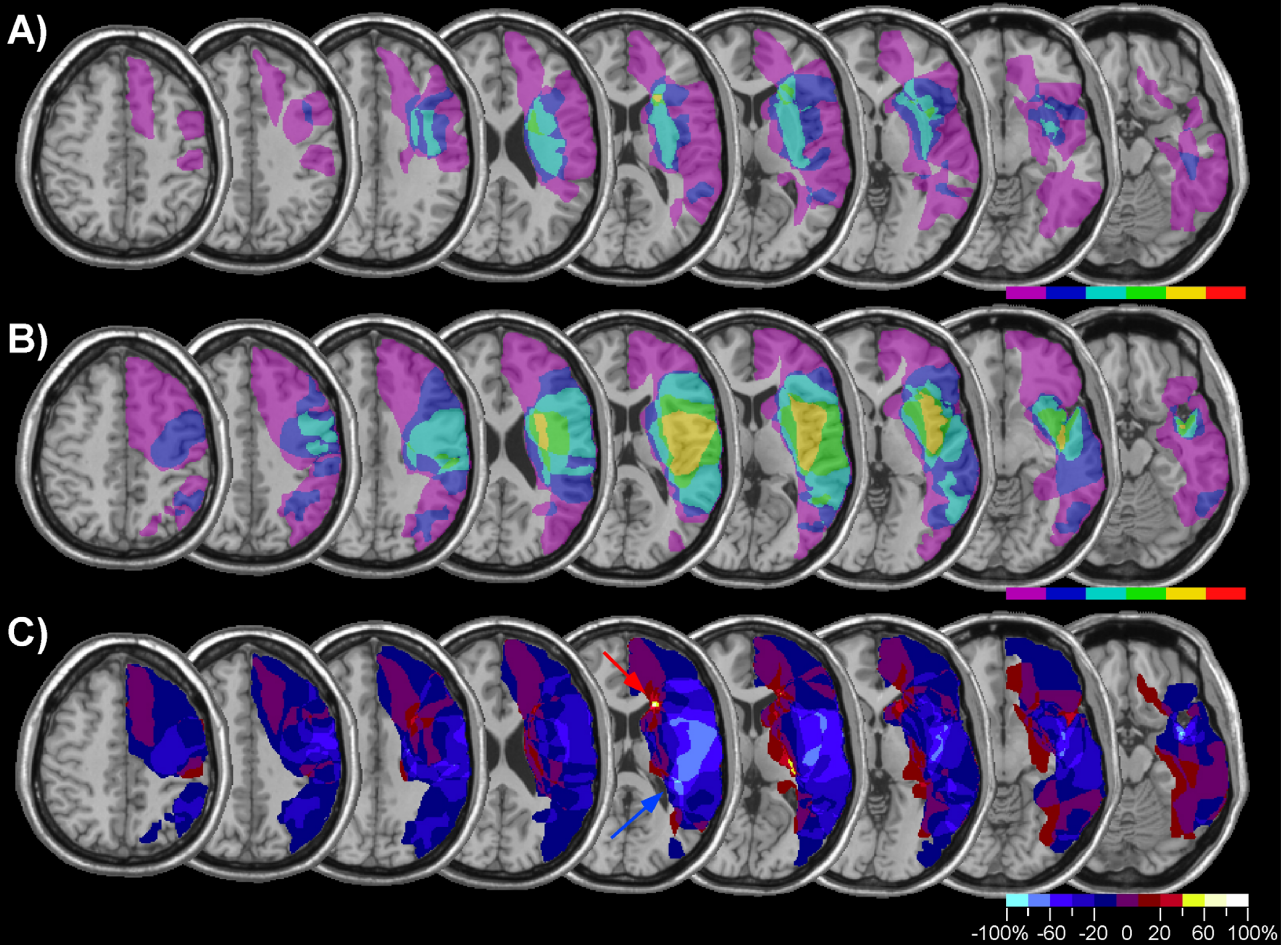
**Anatomical bases of cancellation and line bisection performance**. Lesion analysis of 6 patients with spatial neglect showing impaired cancellation, but relatively spared line bisection (A) and 6 patients with neglect showing the reverse pattern (B). The frequency of overlapping lesions is reflected in the colour scale (violet: n = 1; red: n = 6). C) Subtraction map showing relative incidence of damage in patients with particularly impaired cancellation after subtraction of patients with particularly impaired line bisection. Reddish colours indicate more frequent damage in the neglect group identified by impaired cancellation, coded in bins of 20% from dark red (1–20%) to yellow (80–100%). Bluish colours indicate more frequent damage in the neglect group identified by impaired line bisection, from dark blue (1–20%) to light blue (80–100%).

Figure 5A and 5B shows the lesion-density plots for the 'cancellation' and 'bisection' groups, respectively. In the 'cancellation' group five of six patients shared a common lesion, located in the white matter of the frontal lobe, anterior to the caudate nucleus. In the 'bisection' group, five of six patients shared a lesion centred on the insular cortex, comprising the white matter beneath the STG and the paraventricular white matter of the parietal lobe. The subtraction of lesions of the 'bisection' group from the 'cancellation' group (Figure 5C) confirmed that the 'cancellation' group had significantly more frequent damage in the white matter anterior to the caudate nucleus [red arrow; Fishers test: p < .05]. In contrast, damage of the insular cortex and the white matter beneath the TPJ and the posterior STG was more frequent in the 'bisection' than the 'cancellation' group [blue arrow; Fishers test: p < .05].

## Discussion

Using a lesion comparison method previously applied by several authors the present study confirms the findings of previous studies evaluating the anatomy of spatial neglect. Certainly, several aspects of the methodology of our study may be criticized. Thus, lesion analysis based on CT-scans is limited because of the low resolution of CT-images in the z-plane. Though in most patients MRI sequences were acquired, which have better resolution and better sensitivity for cerebral infarction than CT-scans, in some patients only T1-weighted MRI had been performed which does not always differentiate well between intact and injured tissue. In addition, CT- and MRI-scans were acquired for clinical purposes and consequently differed with respect to the orientation of axial slices as well as the imaging protocol. Although we tried to minimize these problems as possible by including only those patients in whom damaged brain tissue could clearly be identified, they might have influenced the lesion mappings.

Considering these difficulties it is remarkable how consistent our results are with the findings of previous studies. When comparing the lesions of chronic patients carefully selected for the presence of behavioural and test criteria of spatial neglect with non-neglect patients, we identified three sites that were significantly more often damaged in neglect patients: a region centred around the TPJ reaching into the supramarginal gyrus and the posterior STG; the white matter anterior to the caudate nucleus; and the insula. Several older [7,8] and more recent [11,14] studies have reported that damage to the TPJ and the IPL involving the supramarginal and angular gyrus is a frequent cause of spatial neglect. Neuropsychological and functional imaging studies suggest that the TPJ is involved in covert or overt orienting of attention away from ipsilateral stimuli: the so-called disengagement deficit of attention [28-30]. Damage to this region is therefore the most likely neuroanatomical correlate of the impaired ability of neglect patients to disengage attention from ipsilesional distracters [31,32].

The second region that was significantly more often damaged in neglect compared to non-neglect patients was the posterior insular cortex. Spatial neglect after isolated insular lesions has occasionally been reported [33], and frequent involvement of the insula has been found in previous anatomical studies of spatial neglect [15,34]. The insula has reciprocal connections to the inferior parietal and lateral prefrontal cortex and is particularly involved in somatosensory and auditory processing [35]. Therefore, insular damage may be responsible for neglect symptoms affecting primarily non-visual modalities, such as disorders of body exploration or tactile extinction [33].

While brain tissue surrounding the TPJ and insular cortex were reliable predictors of spatial neglect in our study, the association of the frontal white matter rostral to the caudate nucleus with spatial neglect was less reliable. This region was very small and was significantly more often damaged in neglect than non-neglect patients only when patients with haemorrhagic injury were included. Thus, though damage to caudate-frontal projections might lead to hypoperfusion of the prefrontal cortex [36] the importance of the frontal white matter lesion must be considered with caution.

Our results are of particular relevance in view of the recent controversy concerning the brain region whose damage is critical for the occurrence of spatial neglect. While Mort and collaborators [14] identified the IPL and in particular the angular gyrus as the lesion site that is most frequently associated with spatial neglect, Karnath et al. [15] reported that damage to the middle STG differentiated best between neglect and control patients. In the present study the critical region was centred on the TPJ and extended superiorly into the supramarginal gyrus and anteriorly into the posterior STG. However, we found that though sensitivity of IPL and STG damage were comparable, specificity of IPL damage as a predictor of spatial neglect was significantly higher than of STG damage. In other words, damage to the IPL is highly predictive of the occurrence of spatial neglect while the STG is often damaged in patients with neglect, but also more often than the IPL in patients without neglect.

Several factors might explain why the critical lesion associated with spatial neglect was localized more anteriorly in the study of Karnath et al. [15] compared to the study of Mort et al. [14] and our own. Karnath et al. [3] suggested that the inconsistencies between findings of anatomical studies might reflect the fact that some neglect patients were included on the basis of cancellation performance whereas in others neglect had been assessed on the basis of biased line bisection. Indeed, several studies suggest an anatomical dissociation between cancellation and line bisection performance [19,26,27,37], a finding confirmed by our lesion comparison of two distinct neglect subgroups differentiated on the basis of their disturbed cancellation and line bisection performance. However, though these studies all show that greater line bisection error is associated with posterior damage, the lesion overlap is still very variable. In addition, the present results [as well as those of Binder et al., 19] suggest that impaired cancellation performance in patients with relatively intact line bisection is associated with deep frontal damage rather than damage to the STG as would be suggested by the hypothesis of Karnath et al. [3]. It is therefore uncertain whether this hypothesis might explain the divergent findings.

An alternative possibility is that differences in patient selection resulted in contrasting anatomical findings. The patients of Karnath et al. [15] were examined 8 days post-stroke compared to 63 days in the study of Mort et al. and 57.5 in the present report. In a recent large-scale study Ringman et al. [17] reported that about five sixth of the patients showing severe neglect seven days post-stroke recovered within three months. Thus, a considerable number of neglect patients examined within one week post-stroke would be expected to recover relatively rapidly from their acute symptoms. A tentative hypothesis emerging from this finding is that damage to the STG may result in neglect symptoms that recover rapidly, but that only the combination of STG and IPL damage results in chronic neglect.

Finally, it is appealing to consider the controversy about the critical brain damage leading to spatial neglect in light of the hypothesis that neglect might result from intra-hemispheric disconnection. A recent study using electrical stimulation of patients undergoing surgery of right temporo-parietal glioma reported that stimulation of the superior longitudinal fasciculus (SLF) resulted in greater line bisection errors than stimulation of the right IPL or posterior STG [38]. The SLF connects the inferior parietal lobe with dorsolateral prefrontal cortex and runs through the white matter beneath the STG. As discussed in a recent review of neuroanatomical studies of spatial neglect [39], the regions of maximal overlap identified in the studies by Mort et al. [14] and Karnath et al. [15] are both compatible with a fronto-parietal disconnection. Likewise, the voxel-wise analysis in the present study identified a region that reaches into the white matter beneath the TPJ and the STG and thus may disrupt fibertracts connecting the inferior parietal lobe with the prefrontal cortex.

## Conclusion

Neurophysiological, neuropsychological, and functional imaging studies indicate that spatial attention is subserved by a distributed network of cortical and subcortical regions involving the IPL, STG, prefrontal cortex, and the insula [28,29,40]. In agreement with these findings, we found that damage to the IPL, posterior STG, the insula and parieto-frontal projections was most predictive of chronic spatial neglect. These results suggest that only extensive damage or disconnection of the network for spatial attention may lead to a severe and chronic neglect syndrome.

## Competing interests

The authors declare that they have no competing interests.

## Authors' contributions

LG and RP acquired clinical data and performed the lesion analyses; RP analyzed the data; LG, RP and AS drafted the manuscript. All authors read and approved the final manuscript.
